# Challenges of recruiting emergency department patients to a qualitative study: a thematic analysis of researchers’ experiences

**DOI:** 10.1186/s12874-020-01039-2

**Published:** 2020-06-11

**Authors:** Delyth Price, Michelle Edwards, Andrew Carson-Stevens, Alison Cooper, Freya Davies, Bridie Evans, Peter Hibbert, Thomas Hughes, Tim Rainer, Niro Siriwardena, Adrian Edwards

**Affiliations:** 1 Division of Population Medicine, Cardiff University School of Medicine, Cardiff, Wales; 2 Swansea University Medical School, Swansea University, Swansea, Wales; 3 Centre for Healthcare Resilience and Implementation Sciences, Australian Institute of Health Innovation, Macquarie University, Sydney, Australia; 4 Emergency Department, John Radcliffe Hospital, Oxford, England; 5 Community and Health Research Unit, School of Health and Social Care, University of Lincoln, Lincoln, England

**Keywords:** Patient recruitment challenges, Qualitative research, Emergency department, Patient experience

## Abstract

**Background:**

At times of increasing pressure on emergency departments, and the need for research into different models of service delivery, little is known about how to recruit patients for qualitative research in emergency departments. We report from one study which aimed to collect evidence on patients’ experiences of attending emergency departments with different models of using general practitioners, but faced challenges in recruiting patients. This paper aims to identify and reflect on the challenges faced at all stages of patient recruitment, from identifying and inviting eligible patients, consenting them for participation and finally to engaging them in interviews, and make recommendations based on our learning.

**Methods:**

A thematic analysis was carried out on field-notes taken during research visits and meeting minutes of discussions to review and improve patient recruitment throughout the study.

**Results:**

The following factors influenced the success of patient recruitment in the emergency department setting: complicated or time-consuming electronic health record systems for identifying patients; narrow participant eligibility criteria; limited research nurse support; and lack of face-to-face communication between researchers and eligible patients.

**Conclusions:**

This paper adds to the methodological evidence for improving patient recruitment in different settings, with a focus on qualitative research in emergency departments. Our findings have implications for future studies attempting to recruit patients in similar settings.

## Background

Increasing demand on emergency departments has led to the development of different models of service delivery [1]. Qualitative research in emergency departments is crucial for understanding patients’ experiences and improving patient-centred care, and research is urgently needed to understand the outcomes of these new service models for patients [2]. However, there is little evidence in the literature about effective patient recruitment for qualitative research in emergency department settings [3].

Challenges to patient recruitment and engagement in research can occur at all stages of the process: initially identifying and inviting eligible patients; gaining their consent; and successfully engaging them in data collection [4]. The unscheduled nature of emergency department visits poses specific recruitment challenges, due to the urgent nature of emergency department patient conditions and the demanding work environment that the emergency department presents [5]. Limited success in identifying and inviting eligible patients for research has been associated with poor collaboration between hospital sites and researchers [4]. Hospital staff may have doubts about their involvement in the research if they are unsure of the purpose of the research or have concerns about their level of skill in identifying and inviting eligible patients [6]. Furthermore, narrow eligibility criteria may also contribute to insufficient numbers of patients being identified and invited to participate in research [7].

Once approached to take part, patient participation in research studies is an individual choice based on several factors, such as the purpose of the study, what participation involves, how the findings will be used and who will benefit from the findings [3]. Impersonal communication between researchers and patients (such as postal/ email invitations, or telephone interviews) can hamper attempts to recruit patients, whereas sitting down with a patient and their family to explain the research conveys trust and openness, increasing the likelihood of patients engaging with a study [3, 5, 8]. However, there are practical difficulties associated with obtaining verbal consent in the emergency department setting due to both the nature of the patient’s condition and the busy environment [5]. Telephone reminders to patients following postal invitations have been found to increase patient recruitment [9]. While payment for participation in research is highly debated and can be viewed as presenting ethical challenges [4], a lack of monetary recognition for participation in research has also been identified as a reason for low recruitment rates [5, 8]. Despite these significant challenges in recruiting patients in emergency departments for research, obtaining patient perspectives on their care and experiences in strained settings such as the emergency department are crucial to improving patient care and safety [2].

The literature demonstrates a range of potential barriers and solutions to recruiting patients for research. However, existing research has generally focussed on patient recruitment into large clinical trials, as opposed to smaller-scale qualitative research in defined care settings. We aim to describe the factors which influenced patient recruitment in our study of different general practitioner models in the emergency department setting, by exploring the key challenges and consequent amendments made to our processes. From our learning, we propose recommendations for future research that seeks to recruit emergency department patients for qualitative research.

## Methods

### Context: setting

Our UK National Institute for Healthcare Research, Health Services and Delivery Research (NIHR DS&DR) funded study, “GPs in EDs” [10], recruited 13 hospital emergency departments in England and Wales as “case study sites”, whereby mixed methods were used to carry out an in-depth evaluation of the effectiveness of different models of general practitioners working in or alongside emergency departments. Three of these 10 case study sites did not have any model of using general practitioners in their emergency department and acted as “control sites” for comparison. We included patient data for this analysis from one further site which had initially been planned as a case study site, but following the research visit it was decided that it did not meet the eligibility criteria for all data to be included in the overall study.

One key objective was to understand the impact on patient experience, by conducting qualitative interviews with patients. Much consideration was given to effective recruitment methods for this qualitative research element, particularly in a hard-to-reach area such as an emergency department. In line with best practice, public and patient members are study Co-Applicants alongside multi-disciplinary and specialist partners [11]. Two public members are also members of our independent Study Steering Committee and have worked alongside stakeholder and academic colleagues to oversee study implementation. All colleagues contributed to decisions throughout the research about enhancing data collection through patient interviews.

### Patient recruitment methods in the GPs in EDs study

We aimed to recruit a purposive sample of 60–120 patients from our case study sites, with specific conditions appropriate for management by either emergency department clinicians or general practitioners working in the emergency department setting. We planned to conduct semi-structured telephone interviews, of 20–30-min duration, following the patients’ emergency department visits to explore patients’ reflections on their experiences. During the early phase of our study at a stakeholder event, a group of expert clinicians, policymakers, public contributors and researchers took part in a consensus exercise to choose five “marker” conditions which were deemed the most suitable for a comparative analysis. These marker conditions comprised of a presenting complaint (e.g. back pain) with associated exemplar diagnosis (e.g. sciatica) with a low acuity score (see Appendix). The aim of patient interviews was to understand how patients with certain symptoms or diagnoses were managed in the different services by different staff, in terms of use of acute investigations, observation times and referral to other acute hospital services. We aimed to recruit 5–10 patient participants at each participating case study site, covering the range of marker conditions. Our initial strategy included two recruitment methods to invite patients into the study, discussed below.

Before agreeing to be included as a case study site, departments stated that they were able to support our research, which included the capability and capacity to support patient recruitment. However, due to the demanding setting, researchers often had to negotiate the level of support for each aspect of the study, offering flexibility if departments were unable to give the ideal level of support needed for identifying and inviting patients. It was important that we were flexible with what we requested of departments, as we needed to recruit sufficient and appropriate departments as case study sites and collect a range of data via different research methods for all aspects of the study (e.g. observations and staff interviews).

#### Initial recruitment method 1: inviting patients via post

The first method aimed to recruit patients via postal invitations sent out by a member of hospital staff (i.e. a NHS-appointed delivery research nurse), who would use the electronic health record (EHR) system to identify up to 50 patients who had attended the case study site emergency department in the last 3 months, with the marker conditions in our recruitment framework (see Appendix). Eligible patients were mailed a patient study pack containing: a study invitation letter, a participant information sheet, two consent forms and a stamped and addressed envelope. The patient information sheet asked patients to sign and return their consent forms to the university if they wished to take part in a telephone interview. Once these were received, the study team would contact the patient by telephone to arrange an interview.

#### Initial recruitment method 2: inviting patients at the emergency department

The second method aimed to recruit patients during the research visit in the case study site emergency department. A member of hospital staff (i.e. a research nurse) would identify up to 50 eligible patients when they were in the emergency department, informing them of the study and providing them with the patient study pack. Patients would be asked to take home the materials they had been given and consider whether they would like to take part, returning the consent form (to the university) if they wished to participate. Once consent forms were received by the study team, the same process for contacting patients would be followed as method 1.

### Amendments to patient recruitment processes

Due to low initial patient recruitment figures from both methods, the study team sought advice from study co-applicants, study steering committee members and public and patient involvement representatives, on potential changes to the recruitment methods. The following amendments were made in May 2018 to help with patient recruitment:

- Inviting up to 100 participants at each case study site rather than 50, to increase returns.
- Printing study invitation letters on hospital headed paper rather than Cardiff University headed paper so that patients were familiar with the sender.
- Giving participants a quicker and easy option of registering their interest by texting ‘yes’ to a mobile number, to then be followed up with a phone call from a member of the research team to request the consent form and arrange an interview.
- Specifying the desired marker conditions on the participant information sheet so that patients could recognise their eligibility for the study.
- Offering an incentive of a £20 high-street shopping voucher to participants who were interviewed.

### Methods of analysis of patient recruitment methods

After identifying problems with patient recruitment, a thematic analysis [12] was used on two data sources to analyse the processes, procedures and experiences of recruiting patients into the GPs in EDs study, to understand reasons for low patient recruitment. The data sources came from study data (researchers’ field notes from research visits to case study sites) and documents from a range of study meetings (progress reports and meeting minutes).

#### Data sources

##### Field notes

The researchers (ME and AC) each produced one set of field notes at each case study site, totalling 26 sets of in-depth field notes. Research visits lasted 2 to 3 days at each case study site, the purpose of which was to gather information about the process of presentation, triage, assessment, investigation/ treatment/ referral, discharge review and waiting times in each hospital, through interviews with staff and field notes. Research visits were also intended to facilitate both methods of patient recruitment: to liaise with members of staff to arrange for patients to be invited via post (method 1), or to invite patients in the emergency department at the time of the research visit (method 2). Field notes were taken throughout each day and as well as capturing the data above, contained reflections on the practical difficulties of the research visit, such as recruiting patients.

##### Progress reports and meeting minutes

Data came from weekly progress updates over the course of 16 months and minutes from 12 study co-applicant meetings; 3 study steering committee meetings and; 4 patient and public involvement meetings. Data included progress updates on patient recruitment figures (numbers of patients invited, consented and subsequently interviewed), experiential anecdotes from researchers about the process of recruiting patients, suggestions from colleagues regarding the reasons for low recruitment and possible ways to improve recruitment.

#### Data analysis

Using NVivo 12 (QSR International V.12), a thematic analysis framework [12, 13] was initially established by coding field note data into themes and sub-themes relating to practical challenges faced by the researchers in recruiting patients at case study sites. This coding framework was applied to the data from progress reports and meetings minutes, and was subsequently expended to include themes and sub-themes relating to the processes and progress of recruiting patients, and the suggested changes to patient recruitment from colleagues. Throughout the thematic analysis, themes were reviewed, modified and developed [12, 14] and meetings were held with both researchers to validate themes.

These thematic analyses allowed us to understand the challenges facing patient recruitment for qualitative research in the emergency department setting, the process and progress of recruiting patients throughout the study via different research methods, and the suggested changes to recruitment procedures made by colleagues. These analyses can inform recommendations for future research.

## Results

### Total number of patients invited, consented and interviewed

In total, 748 patients were invited to take part in a telephone interview for the study, 43 (6%) patients consented and 24 (3%) were subsequently interviewed, with 19 patients either withdrawing or being uncontactable for interview after consenting to take part. We successfully recruited patients at nine case study sites. Following the first research visit to a ‘control’ site (hospitals without a model of using general practitioners at the emergency department), the decision was made not to recruit patients at control sites, therefore no patients were recruited at the following two control sites. We were unable to recruit patients from two case study sites because of limited staff availability (such as research nurses, clinicians or administrators) and local research and development protocols which restricted data sharing. The number of patients invited via both methods at each case study site is shown in Table 1.

**Table 1:** Total number of patients invited via both methods

Case study site identification number	Patients invited via post (method 1)	Patients invited in-person (method 2)
**GPED02**	50	0
**GPED03**	160	1
**GPED04**	191	1
**GPED05**	50	4
**GPED06**	90	0
**GPED07**	0	0
**GPED08**	0	0
**GPED09**	39	0
**GPED10**	100	0
**GPED11**	50	4
**GPED12**	0	0
**GPED13**	0	5
**GPED14**	0	3
**GPED15**	0	0

The amendments made to improve patient recruitment did not have a notable impact on patient recruitment. Overall, 304 patients were invited before the amendments, resulting in 5 interviews (2%) and 444 patients were invited after the amendments, resulting in 19 interviews (4%). Table 2 shows the number of patients interviewed from each marker condition.

**Table 2:** Number of patients interviewed for each marker condition

**Breathlessness**	**Back pain**	**Abdominal pain**	**Febrile child parent/ guardian**	**Chest pain**
9	5	4	3	3

#### Patient recruitment for method 1

**Fig. 1:**
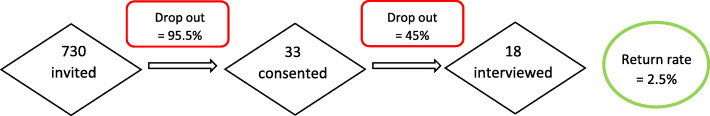
Recruitment for Method 1

Some case study sites were unable to allocate research nurse support to invite patients to the study via post (*n* = 6), and we could not use this recruitment method at these sites. From those case study sites that were able to assist with inviting patients by post (*n* = 8), Fig. 1 shows the number of patients who were invited, consented and interviewed:

#### Patient recruitment for method 2

**Fig. 2:**
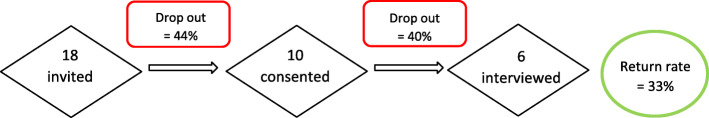
Recruitment for Method 2

Many case study sites (*n* = 8) were not able to invite patients in the emergency department during the research visit. From those case study sites who were able to invite patients in the emergency department (*n* = 6), Fig. 2 shows the number of patients who were invited, consented and interviewed:

Despite the small sample size for recruitment method 2, the success rate was higher with recruitment method 2 (inviting patients in person; *n* = 33.3%) than method 1 (inviting patients via post; *n* = 2.5%).

### Findings following thematic analysis

The following themes were identified as contributing to low patient recruitment in the GPs in EDs study: complicated or time-consuming electronic health record systems; narrow eligibility criteria; limited research nurse support; and lack of face-to-face communication between researchers and patients. These themes are described in Tables 3, 4, 5, and 6, with suggestions to improve recruitment for future research.

**Table 3:** Complicated or time-consuming electronic health record systems

Findings	Evidence	Suggestions for future research
In some departments, it was difficult to identify patients using the electronic health record (EHR) systems in place. Some departments used multiple systems for different areas of the department (e.g. registration, triage assessment, discharge notes etc.), meaning that all systems had to be looked at separately to identify eligible patients. At one hospital, the EHR system was not set up to retrieve data by specific details such as presenting complaint, and so the task of identifying eligible patients was too difficult. As a result, no patient recruitment could take place at that case study site. Often, EHR systems were very slow and identifying even a small number of eligible patients took much longer than anticipated, for example due to having to switch between multiple EHR systems. This slowed down the process of identifying and therefore inviting patients via both recruitment methods.	*[ED Consultant] informed me that the computer system does not enable them to pull up details by presenting complaint... She seemed to think that there are IT problems and it would not be easy to pull up a list of patients to send invitations to... [she] did not seem to have a lot of enthusiasm for another visit or to find a way of identifying patients on their system.* - (Field notes - hospital 12) *I spent from 10 am - 1.15 [pm] with the research nurse searching for patients on the Maxims system … After over three hours of looking through the system to screen for eligible patients we had only found 13*. - (Field notes - hospital 3)	Implementation of the new Emergency Care Dataset (ECDS) in England, with the intent to extend into Ambulance and Integrated Urgent Care will ensure that in future there will be improved quantitative data to identify both presenting conditions and outcomes in patients who access Urgent and Emergency Care services.

**Table 4:** Narrow eligibility criteria

Findings	Evidence	Suggestions for future research
Because of the narrow eligibility criteria, and the need to identify eligible patients by a specific diagnosis associated with the presenting complaint, often it was difficult to identify sufficient numbers of eligible patients, particularly for recruitment method 2 during the research visit (see Appendix), for example if the diagnosis was made at the end of the patients’ visit. Some emergency departments did not see children or streamed them to a separate paediatric assessment unit at the hospital, rather than the GP service in the emergency department. Furthermore, public health education has encouraged patients with chest pain to phone an ambulance. For those who do self-present in the emergency department, many departments had strict guidelines which meant chest pain patients were automatically seen by an emergency department doctor. Thus, local protocols made it difficult to identify children who had been seen in the emergency department and patients with chest pain who had been seen by a general practitioner.	*There were not enough patients coming through the department with marker conditions during the time we were there. I couldn’t find one patient on Saturday afternoon.* - (Field notes - hospital 4) *We did not find any [patients] who had seen a GP with chest pain as they usually go to ED doctor.* - (Field notes - hospital 9)	While all research needs appropriate eligibility criteria to answer its research question(s), consideration should be given to how eligible patients will be identified. Using broader initial eligibility criteria (for example, just searching by presenting complaint rather than presenting complaint and diagnosis) may result in more patients being identified.

**Table 5:** Limited research nurse support

Findings	Evidence	Suggestions for future research
Research nurse support was needed not only to identify eligible patients, but also to prepare and post the research packs (for method 1) or approach eligible patients on behalf of the researchers (for method 2). While the study team conducted as much of the research pack preparation as possible, labelling and distributing the packs could only be carried out by staff at the hospital sites for both recruitment methods, due to data protection guidance and ethical approvals of processes. One department stated that they were more likely to allocate their research nurses to larger studies which brought in more patient participants. The availability of support from research nurses or other staff in the department to help facilitate recruitment was varied. We could invite and therefore interview many more patients (via both recruitment methods) in those departments where a research nurse or other staff member was able to offer support than in those where one was not. Research nurses greatly influenced the success of a research visit.	*Having a research nurse with us for 3 days was invaluable.* - (Field notes - hospital 3) *At one-point, reception told me that there were some patients waiting to go to minors, but we could not approach them and we could not find anyone to assist us to approach them. We did not have a research nurse to be able to approach patients on our behalf.* - (Field notes - hospital 14)	Good contact before the visit helps to inform and prepare staff members for the research visit, improving their understanding of the study and how they might help. If practical preparations (such as preparing patient packs) can be carried out in advance of a research visit, this can lessen the burden on research nurses involved in the study, thus generating more willingness to offer support to research.

**Table 6:** Lack of face-to-face communication between researchers and patients

Findings	Evidence	Suggestions for future research
Our patient and public involvement representatives felt that patient wariness was a likely reason for low patient recruitment. For example, patients could be wary about taking part in an interview which might ask them to justify their reasons for seeking emergency care, especially if they were aware that they had been seen by a general practitioner, or were told by a clinician that their condition could have been managed more appropriately by their own general practitioner. Our co-applicants and patient and public involvement representatives also considered how face-to-face recruitment makes the research more memorable for the patient, thus improving recruitment and retention. Because most patients were invited by post, there was little opportunity for researchers to reassure patients about the purpose of the interviews. This lack of face-to-face communication may have resulted in fewer patients recruited.	The total returns rate for face-to-face invitations (method 2) was 33%. In contrast, the total returns rate for postal invitations (method 1) was 2.5%.	While it may take more time and require more complex ethical approvals, future research should consider research designs which utilise face-to-face recruitment methods, for example using more informal interviewing methods. Further consideration should be given to the process of consenting patients. Ensuring that this is a smooth process, for example by allowing patients to consent during their time in the department, rather than requiring consent in the post, may improve patient recruitment.

## Discussion

### Principal findings

We found that the following interdependent and interacting factors contributed to low patient recruitment for this qualitative study in the emergency department setting: complicated or time-consuming electronic health record systems; narrow eligibility criteria; limited contact with/ availability of research nurses or other support staff; and a lack of face-to-face communication between researchers and patients. Amendments made to our methods did not substantially improve recruitment: 2% of patients invited before amendments were interviewed and 4% of patients invited after amendments were interviewed.

Narrow eligibility criteria limited the number of patients who could be identified and invited. This made searching for eligible patients on already complicated electronic health record systems even more time consuming. Both factors made our recruitment processes time consuming, meaning research nurses could not always commit enough time to supporting patient recruitment, making engagement in patient recruitment less feasible for case study sites. Limited availability of research nurses was also due to the small size of our study, as departments are more likely to allocate resources to large clinical trials and commercial studies where more patients will be recruited and therefore more accruals (monetary credits) will be obtained. These factors all interacted to limit the number of patients identified and invited to take part in the study.

In terms of recruiting patients once they had been invited, we believe that a lack of face-to-face communication between researchers and patients meant that invitations to participate were impersonal, easily ignored and could lead to patient wariness about participation.

### Strengths and limitations

The experience reported in this paper is helpful for understanding the reasons for low patient recruitment in the GPs in EDs study and other studies in similar settings. By using qualitative methods of evaluation to analyse meeting minutes, in-depth field notes and recruitment methods, figures and amendments, this paper gives an insight into the reasons for low patient recruitment and highlights ways to improve patient recruitment in future studies using a similar setting. However, we were unable to obtain data from those who did not respond to invitations or consented but then declined, to explore these patients’ reasons for not participating. The findings in this paper are based on one study’s experiences and further evidence is needed.

### Context of other literature

The findings from this paper fit with the current literature surrounding patient recruitment in research, but highlight the need for further research into patient recruitment for qualitative research in the emergency department setting. Studies have found that narrow eligibility criteria can restrict patient recruitment, as this limits the number of patients who can be invited and thus interviewed, as well as slowing down and often complicating the process of identifying eligible patients [7]. This is consistent with our experience in the GPs in EDs study, as it was often difficult to find suitable numbers of eligible patients within time constraints available to us, and broadening the eligibility criteria would have led to more patients being invited and therefore recruited [7].

Studies have identified that if preparatory work (e.g. helping to screen and identifying appropriate patients, preparing appropriate recruitment materials, informing relevant staff members about the study) can be carried out by study members (e.g. research staff, study support staff), then the burden on hospital staff is lessened, which can be key to ensuring successful engagement of staff and recruitment of patients [4]. Furthermore, having other priorities and not having much time to dedicate to a study is a known barrier to hospital staff being able to help with patient recruitment [6]. In the GPs in EDs study, support of research nurses or other staff at hospitals was key to identifying and inviting appropriate numbers of patients, and thus patient recruitment was highest at those case study sites which were able to allocate the most support. If higher levels of support had been available at all case study sites, then the GPs in EDs study may have achieved higher patient recruitment numbers. This may itself have been achieved through further preparatory work by the research team before a research visit, or hospital staff being allocated additional time for recruitment during a visit.

Face-to-face communication is valued by research participants and informing patients (and their family members) of the research in person allows rapport to be built, in an open and trustful manner, and can increase the likelihood of the patient engaging in the research [3, 8]. Again, this is supported by our experiences, as we found higher participation among patients invited in person rather than by post, albeit with a small sample size. The wider body of literature, however, recognises the difficulty of face-to-face communication between researchers and patients in emergency care settings, as in the GPs in EDs study, particularly due to the high demand and business of emergency departments and the unscheduled nature of patient attendance to emergency departments [5]. While the using telephone reminders may have increased patient recruitment [9], the ethical implications of contacting patients via telephone prior to the patient giving consent to be contacted would need serious consideration.

Previous research has successfully used “informal interviewing” as a practical technique for gaining patient perspectives, in person, in busy emergency departments [15]. Informal interviewing involves informal conversations with participants to enable more open discussions than formal interviewing, making the process of gathering data on patient experience easier and faster than formal interviewing methods [15].

### Future research

While we have been able to identify key factors which restricted the ability to identify and invite patients into the study, challenges were faced in terms of identifying patients’ reasons for declining participation once invited. Future research could explore emergency department patients’ possible reasons for not taking part in research, to develop patient recruitment methods that encourage participation. Furthermore, the learning from this paper comes only from one study’s experiences and could be formally evaluated in further larger studies or clinical trials. Further research is also needed into how researchers can best work with patient and public involvement representatives to increase patient recruitment in different settings [16].

## Conclusion

This paper adds to the methodological evidence for improving patient recruitment in different settings, with a focus on qualitative research in emergency departments. We found that patient recruitment in the emergency department setting was influenced by slow or time-consuming electronic health record systems, narrow eligibility criteria, the support (or lack of support) from research nurses or other staff member; and lack of face-to-face communication between researchers and patients. These findings can be used to inform methods planned by researchers attempting to recruit emergency department patients for future qualitative research.

## Data Availability

The datasets used and/or analysed during the current study are available from the corresponding author on reasonable request.

## Appendix

### Patient Recruitment Guide

#### Patient recruitment pathway

Please use the table below to help us identify 50 patients in total – 10 for each marker condition (5 seen by GP, 5 seen by ED clinicians).

#### Guidance for selecting patients to invite to interviews

- Please can you use the patient recruitment guide to find us 50 patients with the listed chief complaints that have a diagnosis listed against that complaint (it’s really important that we only have a diagnosis that is listed in the column). If you have capacity to look for more patients we can send out up to 100 letters if the response rate is low. We can initially send out 50 and then send more if we need to.
- Before the visit, we will email you our ‘study invitation letter’ which will need to be printed on your hospital’s headed paper
- When you receive our patient packs, please add the study invitation letter to the front of the packs, with the patient name written at the top of the letter.
- We need to send letters to five patients with each complaint and matching diagnosis that have been seen by a member of the primary care team (GP or ANP) please ask the ED to provide you with a list of GPs or ANPs that are working in the primary care service.
- We also need to send letters to five patients for each complaint that have been seen by an ED doctor or ENP. It will be useful to know who the ENPs are and who the doctors are too.
- We only need to know the type of practitioner that saw the patient, we do not need to know their name.
- We are looking for patients who have visited the ED in the last 3 months
- We are aware that some of our complaints might not be seen by many GPs e.g. chest pain so it might be difficult to find enough patients
- If acuity scores are used we are looking for patients who score 3,4 or 5 (not 1&2 so not patients who have life threatening symptoms)
- We have excluded patients where it is noted that they may have serious mental health problems and dementia. We have also excluded patients who do not speak English as a first language.
- We try to get a mix of gender and ages (we do not have any upper age limit on adults, for children with a fever we want children under 10).
- Please record details of patients on the spreadsheet and email back to us – It might be useful for you to keep another version for yourself with more patient details on e.g. address and patient ID
- **Michelle can meet with you to help get started with screening and help with organising the letters to get sent out.**
- **If you have any questions please contact Michelle Edwards by email- Edwardsm28@cardiff**

**Table:**

Marker condition	Time period	ECDS Acuity	Chief complaint	Patient age	Exemplar diagnoses	Seen by
Child < 10 with a fever	Last 3 months	3,4,5	Fever	Less than 10	Infectious disease Respiratory Upper respiratory tract infection Surgical ENT Otitis media/ear infection Surgical ENT Tonsillitis	5-10GPs 5–10 ED staff
Cough and breathlessness	Last 3 months	3,4,5	Short of breath Difficulty breathing Noisy breathing Coughing up blood	Any	Infectious disease Respiratory Lower respiratory tract infection Infectious disease Respiratory Bronchopneumonia Infectious disease Respiratory Lobar pneumonia	5–10 GPs 5–10 ED staff
Abdominal pain	Last 3 months	3,4,5	Abdominal pain	Any	Infectious disease GU/GI Infectious gastroenteritis Infectious disease GU/GI Urinary tract infection	5-10GPs 5–10 ED staff
Back pain	Last 3 months	3,4,5	Pain in back/trunk (no injury)	Any	Soft tissue injury/wound Muscle injury Lower back Soft tissue injury/wound Sprain/ligament injury Lumbar spine Musculoskeletal Orthopaedics Sciatica	5–10 GPs 5–10 ED staff
Chest pain	Last 3 months	3,4,5	Chest pain	Any	Medical Gastroenterology Oesophageal spasm Medical Gastroenterology Gastro-oesophageal reflux Medical Gastroenterology Gastritis Musculoskeletal Rheumatology Costochondritis Medical Respiratory Pulmonary embolism	5–10 GPs 5–10 ED staff

## Abbreviations

ED: Emergency department
EHR: Electronic health record
GP: General practitioner
GPs in EDs: General Practitioners in Emergency Departments
HS&DR: Health Services and Delivery Research
NHS: National Health Service
NIHR: National Institute for Health Research
